# Integration of Destructive and Non‐Destructive Analytical Determinations for Evaluating Quality of Fresh and Roasted Hazelnuts Subjected to Different Processing Temperatures

**DOI:** 10.1002/fsn3.70095

**Published:** 2025-03-12

**Authors:** Riccardo Riggi, Margherita Modesti, Gianmarco Alfieri, Giuseppe Esposito, Paolo Cucchiara, Serena Ferri, Fabio Mencarelli, Andrea Bellincontro

**Affiliations:** ^1^ Department of Innovation in Biological, Agro‐Food and Forestry Systems (DIBAF) University of Tuscia Viterbo Italy; ^2^ Stelliferi & Viconuts SPA Viterbo Italy; ^3^ Department of Agriculture Food and Environment (DAFE) University of Pisa Pisa Italy

**Keywords:** aromatic patterns, chemometrics, hazelnuts, non‐destructive technologies, roasting, VOCs

## Abstract

The internal quality of hazelnuts ( *Corylus avellana* L.), particularly in terms of the degradation of fat components, is widely recognized as a key factor in determining the appropriate type of industrial processing. Additionally, the internal composition and volatile profile of hazelnuts change significantly based on different roasting conditions. The here reported study investigates the efficiency of Electronic Nose (E‐nose) and Near‐Infrared Spectroscopy (FT‐NIR) technologies, combined with multivariate statistical techniques, for the rapid discrimination of hazelnuts subjected to different roasting conditions. Moreover, the study examines the ability of NIR to predict several key quality parameters in fresh and processed hazelnuts. Hazelnut samples were collected throughout the entire industrial processing chain, from delivery to roasting. The influence of three different roasting temperatures (140–150‐160°C) was evaluated, keeping the roasting time constant at 24 min. Partial Least Squares models were computed to estimate moisture content, total soluble solids, protein content, acidity, and peroxide index through correlation with FT‐NIR spectral data. Excellent regression performances were achieved for all quality parameters, except acidity, with correlations ranging between 0.951 and 0.918. Discriminant analysis models, specifically PLS‐DA and Cluster Analysis, were used to assess the ability to discriminate hazelnuts subjected to different roasting conditions using FT‐NIR and the Electronic Nose as non‐destructive tools. Obtained results from these non‐destructive techniques, particularly the volatile characterization GC/MS‐performed, accurately reflected the differentiation of samples observed through traditional chemical analyses, effectively distinguishing different groups of samples based on roasting temperature. The use of non‐destructive tools such as FT‐NIR and E‐nose during the post‐harvest life and processing of hazelnuts offers an excellent solution for monitoring key quality parameters significantly important for the food industry.

## Graphical abstract

Non‐destructive techniques including FT‐NIR spectroscopy and E‐nose detection, combined with chemometric approaches, demonstrated strong potential for real‐time hazelnut quality assessment. FT‐NIR spectroscopy accurately predicted moisture content, while E‐nose differentiated roasting intensities. Their feasibility in process monitoring highlights a significant advantage over traditional, labor‐intensive methods, offering a rapid and efficient solution for quality control in the hazelnut industry.

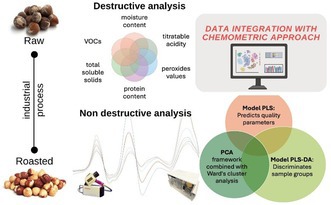

## 1 Introduction

European hazelnuts ( *Corylus avellana* L.) are used worldwide for the confectionery, bakery, and chocolate industries (Pannico et al. 2015; Silvestri et al. 2021) The primary hazelnut‐producing areas are situated between the 40th and 45th latitudes North, but in‐shell production remains concentrated in a few countries, with Turkey (650,000 tons), Italy (102,000 tons), the USA (86,000 tons), Azerbaijan (75,000 tons), Georgia (36,900 tons), and Spain (6700 tons) being major contributors. However, hazelnut cultivation has seen recent expansion and establishment in new locations, including Chile, South Africa, and Australia (FAOSTAT 2023; Molnar and Capik 2012).

From the nutritional point of view, Hazelnuts are a source of bioactive compounds such as phytosterols and vitamin E (Delgado et al. 2010). They are sold both shelled and in shell, and they can be roasted to modify color, flavor, and texture considering the industrial destinations. The pre‐drying process holds significant importance in the postharvest processing of hazelnuts to ensure food safety and quality during storage (Kaveh et al. 2018). Maintaining a recommended safe moisture content of 6% for in‐shell hazelnuts becomes crucial to preserve the quality and to avoid the development of undesirable microorganisms (Wang et al. 2018; Ozay et al. 2008). Raw pre‐dried hazelnuts are often further roasted to improve the color, texture, and flavor (Burdack‐Freitag and Schieberle 2010). The application of heat treatment leads to biochemical changes including the modification of lipid structure and Maillard reactions which promote the production of pyrazine compounds, typically associated with pleasurable roasted flavor (Amaral et al. 2006; Saklar et al. 2001). The essential flavor precursors are free amino acids and monosaccharides, and the accurate control of flavors during roasting is difficult. Traditionally, color can be used as an active indicator because brown pigments increase during the roasting and caramelization processes. However, selecting a roast based solely on color can be misleading, as factors like ripeness, variety, moisture content, and kernel size significantly influence fruit color (Pattee et al. 1982; Sanders et al. 1989). Additionally, hazelnuts, due to their high fat content, are highly prone to lipid hydrolysis and oxidation, both enzymatic and non‐enzymatic. Exposure to light and heat can accelerate these processes, leading to the release of free fatty acids that compromise the stability of hazelnut oil (Fu et al. 2016). Nevertheless, excessive heat during drying or processing can cause significant damage, negatively impacting the flavor, appearance, and edibility of the product. Higher temperatures and longer roasting times lead to a slight increase in oleic and saturated fatty acids, accompanied by a reduction in linoleic acid. Similarly, roasted samples show an increase in triacylglycerols with oleic acid moieties and a decrease in those containing linoleic acid moieties. High‐temperature roasting may also reduce beneficial phytosterols and vitamin E homologues while causing a negligible increase in trans‐fatty acids (Silvestri et al. 2021).

One of the most common methods for roasting nuts is convective heat transfer, performed in hot‐air roasters using continuous or batch systems (Perren and Escher 2013). Conventional roasting of nuts is typically carried out at temperatures between 100°C and 180°C for 10–60 min, depending on the intended commercial use. Temperature modulation is a critical parameter, as it plays a significant role in determining the overall quality of hazelnut products (Donno et al. 2013). The quality parameters of hazelnuts affected by roasting conditions are primarily chemical, involving changes in the lipid fraction (acidity and peroxides), soluble solids, and proteins content due to the Maillard reaction, as well as the formation of characteristic volatile compounds. Traditionally, these parameters are assessed using destructive laboratory methods. However, there is an increasing demand for non‐destructive technologies that enable accurate, fast, real‐time, and reliable quality evaluations, driven by the need for consistent quality assurance in food commodities.

Non‐invasive techniques for hazelnuts are gaining traction, focusing on predicting analytical parameters through optical, acoustic, aromatic, and density traits (Silvestri et al. 2021). Among these, near‐infrared spectroscopy (NIRs) and electronic nose (E‐nose) are emerging as promising non‐destructive tools for food analysis, capable of predicting key parameters such as moisture content, acidity, and the presence of off‐flavors (Silvestri et al. 2021; Modesti, Tonacci, et al. 2022; Cozzolino 2021). Chemometric approaches are essential in correlating non‐destructive measurements with destructive analyses using predictive models computed through statistical validation. Key metrics for validation include the determination index (R^2^), standard error in calibration (SEC), standard error in prediction (SEP), and the ratio performance to deviation (RPD) index. Despite their potential, research on hazelnut post‐harvest quality remains limited compared to other food products. This highlights the need to develop predictive models to ensure superior quality through non‐destructive, reliable, and efficient methods. The final goal is to provide hazelnut producers with practical and effective techniques. In this study, Fourier‐transform near‐infrared spectroscopy (FT‐NIR) and E‐nose were evaluated as alternatives to traditional destructive methods for monitoring post‐harvest hazelnut quality.

## 2 Materials and Methods

### 2.1 Biological Material

Hazelnuts “Tonda Gentile Romana DOP” were harvested in 2022 at the C.P.N. Società Cooperativa Agricola “Cooperativa Produttori Nocciole” and Stelliferi&Viconuts S.p.A cooperative farms both located in Ronciglione, Latium region, Viterbo province, Italy. Hazelnuts were then sampled along the production process, from delivery to roasting, as shown in Table 1. For each production steps, three batches of 5 Kg each were sampled and immediately used for the analysis. The decision to sample at different stages of the production chain was motivated by the industry's need to evaluate the quality profile throughout various processing and marketing phases, aiming to capture the widest possible variability. This approach enhances the accuracy and reliability of predictive models by ensuring they reflect diverse conditions. To explore the impact of roasting on hazelnut quality, three distinct roasting temperature were applied studied, each using a fixed duration of 24 min: low roasting (140°C), medium roasting (150°C) and high roasting (160°C). The hazelnuts were roasted at temperatures within the typical industrial range of 100°C–180°C, with a fixed time of 24 min, chosen based on standard practices to ensure realistic and representative conditions. The process was conducted using an industrial stainless‐steel oven (UORMAR, JUMBO 1000, Vuormar Packaging s.r.l. Correzzo, Italy) with a forced‐air ventilation system powered by a burner.

**TABLE 1:** List of hazelnuts samples with reference code and processing conditions used in the experimental trial.

Sample	Code	Process
Fresh in‐shell	Fresh	As collected, at the time of delivery
In‐shell hazelnuts after drying	In‐Shell	Pre‐dried
Shelled hazelnuts	Shelled	Shelled, pre‐dried, unselected
Selected shelled hazelnuts	Selected Shelled	Shelled, pre‐dried, selected with the removal of evident visual defects
Shelled hazelnuts, selected and low‐temperature roasted	Low Roasted	Shelled, pre‐dried, selected with the removal of evident visual defects, roasted at 140°C for 24 min
Shelled hazelnuts, selected and Medium‐temperature roasted	Medium Roasted	Shelled, pre‐dried, selected with the removal of evident visual defects, roasted at 150°C for 24 min
Shelled hazelnuts, selected and high‐temperature roasted	High Roasted	Shelled, pre‐dried, selected with the removal of evident visual defects, roasted at 160°C for 24 min

From each batch, 200 g of hazelnuts (three repetitions for each sample class) were mechanically pressed for oil extraction. The recovered oil was separated by centrifugation at 11200 g for 5 min, and the oil was stored in a freezer at −18°C until further analysis.

### 2.2 Analytical Determinations

#### 2.2.1 Proximal Analysis

Moisture content (MC) was determined on 75 pre‐dried whole hazelnuts in an oven at 105°C until a constant weight was reached. MC was calculated as the difference (expressed in %) between fresh weight and dry weight according to Gazor and Minaei (2005). Titratable acidity (TA, expressed as % oleic acid), peroxide index (PV), determined by iodometric titration (expressed as millimoles of active O_2_ per kg of oil) were determined on the extracted oils according to official European methods of analysis (Commission Regulation 1991). Protein content (PC) was determined following Kruger (2009) by performing the Bradford assay. Determination of soluble solids content (TSS) was carried out by using a benchtop digital refractometer (ATAGO, Palette PR‐32, ATAGO CO. Ltd., Tokyo, Japan).

#### 2.2.2 Volatile Composition

To analyze the aromatic profile of the different samples, the Solid Phase Microextraction coupled with mass gas chromatographic (SPME‐GC MS) approach was used. 200 g of hazelnuts were ground using an IKA mill (IKA IKA‐Werke GmbH & Co. KG, Germany). 5 g of powdered sample was incubated at 45°C for 30 min in hermetically sealed 20 mL vials to promote volatilization of aromatic compounds. The extraction of the volatiles was carried out for 30 additional min at the same temperature using an SPME fiber (50/30 μm, DVB/CAR/PDMS, 1 cm long; Supelco, Bellefonte, PA, USA). The fiber desorbed the extracted volatiles for 5 min at 270°C. The GC used was the Clarus 680 Gas Chromatograph equipped with a split/splitless injector (PerkinElmer, Waltham, Massachusetts). The volatile compounds were separated on a fused silica column (SupelcoWax, 60 m, 0.25 mm, 0.25 μm), using helium as a carrier gas at a flow rate of 1 mL min. The chromatographic conditions were set as follows: initial temperature 40°C for 5 min; 10°C per min until 120°C; 2°C per min until 180°C; and finally, 10°C per min until 230°C. The compounds were identified using a mass spectrometer (Clarus 500 Mass spectrometer, PerkinElmer, Waltham, Massachusetts) coupled to the GC. Each peak was identified by comparison of spectra in the National Institute for Standards and Technology database (NIST98, Version 2.0, USA) by including only compounds with a similarity of 80% or greater. For the relative quantification of individual compounds, TurboMass software (TurboMass, Version 5.4.2 PerkinElmer Inc., USA, 2008) was used. Finally, the area of each peak was normalized to the sum of all areas of the relative chromatogram to reduce variations caused by fiber (Modesti, Taglieri, et al. 2022). All the analyses were performed in triplicate.

### 2.3 Non‐Destructive Determinations

#### 2.3.1 FT‐NIR Acquisition

Spectral acquisitions were taken using a NeoSpectra FT‐NIR (Fourier Transformed Near Infrared) instrument (si‐ware, CA 9402, USA) and processed using SpectroMOST software (si‐ware, CA 9402, USA). For each thesis, 25 hazelnuts were randomly selected for each replicate, for a total of 75 hazelnuts per class. For pre‐dried hazelnuts, spectra were obtained from the same set of 75 hazelnuts used for MC measurements. This allowed testing the correlation of NIR spectra with analytical measurements for modeling and prediction purposes. Dual spectral acquisitions were conducted on individual hazelnuts, covering the range of 1300 to 2600 nm. These measurements were performed by placing a probe in direct contact with the epidermis of the fruit at two opposite equatorial points. The FT‐NIR spectra acquired in duplicate on the individual hazelnuts were subsequently averaged to yield a single spectrum for each hazelnut. The spectra were acquired in transmittance (T) mode and subsequently transformed to absorbance (A), operating the computation A = log 1/T. The averaged spectra were also processed using spectral transformation, which involved applying a Savitzky–Golay filter in the first derivative with 25points of smoothing. This approach enabled the identification of absorbance peaks and the evaluation of the relative vibrational responses of functional groups and related molecules.

#### 2.3.2 E‐Nose Acquisition

The Electronic nose (E‐nose) used was designed, developed, and assembled at the University of Rome Tor Vergata and is based on an array of 12 quartz microbalances (QMBs). The QMBs were functionalized by seven metal complexes (Mg Co, Cu, Zn, Fe, Mn, Sn) and free base (H2) of 5, 10, 15, 20‐tetrakis‐(4‐butyloxyphenyl) porphyrin (TBPP), free base (H3), copper, phosphorus, and manganese complexes of 5,10,15‐triphenylcorrole (TPC), deposited on the quartz surface by spraying (Capuano et al. 2020). The sensing molecules were synthesized in the Department of Chemical Science and Technology at the University of Rome Tor Vergata. QMB is individually connected to an oscillator circuit, utilizing a temperature‐compensated quartz crystal as a reference to measure oscillator output frequencies with a resolution of 0.1 Hz. Gas delivery is managed through a tubeless embedded pneumatic system featuring a poly (methyl methacrylate) (PMMA) manifold with two inlets and one outlet. This system connects to a miniature diaphragm pump (flow range: 0–200 sccm), a three‐way electronic valve, a proportional electronic valve, and a flow sensor. The instrument is USB‐connected and powered, with data acquisition, instrument functions, and settings controlled through proprietary Matlab software (Capuano et al. 2020). For hazelnut measurement, 5 g of sample was incubated at 45°C for 30 min in hermetically sealed 20 mL vials (equipped with a silicon septum) to promote equilibration of headspace. Subsequently, the headspace was extracted for 90 s using a stream of filtered air and directed into the electronic nose sensor cell. Following each measurement, a pure air stream cleaned the E‐nose for an additional 300 s to establish the reference signal. Sensor signals were determined as the resonant frequency shift between the two steady conditions corresponding to sensors exposed to pure air and the sample. The collective sensor signals form patterns (fingerprints) encoding the overall composition of the headspace.

### 2.4 Statistical Analysis and Chemometrics

Destructive analyses were carried out in triplicate for each sample unit. The data were statistically analyzed through one‐way analysis of variance (ANOVA) and Tukey's post hoc test for multiple comparisons with *p* ≤ 0.05 using GraphPad Prism 7.0 (2022 GraphPad Software, CA). Aromatic profile data, normalized as described above, were auto‐scaled and used for cluster analysis with the Ward method (Euclidean distance) and graphically represented through a Heat‐Map. The autoscaled destructive data were used in a PCA analysis (Principal Component Analysis).

Spectra were transformed into absorbance (log 1/T) and subjected to a Savitzky–Golay (SG) pre‐treatment for calculating first derivative values and smoothing with 25 points. Finally, the spectral performance of FT‐NIR measurements was further evaluated for the construction of predictive regression models, specifically partial least squares (PLS) models for various destructive parameters (acidity, peroxides, soluble solids, total proteins, and moisture content). For the PLS model construction for moisture content, a preliminary outlier exclusion was performed (reducing sample size from *n* = 300 to *n* = 221).

Absorbance spectra, only after being autoscaled, were also employed for PLS discriminant analysis (DA) computation. PLS‐DA involves multivariate regression modeling, which is a suitable chemometric approach for spectral detection. In this context, the detected spectra represent the independent variable (X‐block), while the dependent or descriptive variable (Y‐block) corresponds to the sample categories (Fordellone et al. 2018).

PLS and PLS‐DA performed on spectral detections were cross‐validated through the venetian blind (blind thickness = 1) method; obtained results were graphically represented by scatterplot, scoreplot, confusion matrices, and tables. For both PLS and PLS‐DA, the ideal number of latent variables (LVs) was selected after reviewing the scree plot, a diagnostic tool that allowed for determining the best relationship between observed variables and latent variables, corresponding to the point of error minimization, in calibration (RMSEC) and in prediction (RMSECV), respectively. For the PLS model, the prediction correlation coefficients (R^2^), in calibration (R^2^c) and in prediction or cross‐validation (R^2^cv), and the ratio of performance to deviation (RPD) were also calculated and considered as statistical indexes. For PLS‐DA, the variable importance projection (VIP) scores were calculated and graphically represented in order to show the loading contribution to the sample classification and segregation.

Both the original data from GC–MS analysis and E‐nose detections, after being auto‐scaled, were subjected to cluster analysis (CA) modeling using Ward's method, performed within a Principal Component Analysis (PCA) framework (Granato et al. 2018). CA processing was cross‐validated using leave‐one‐out validation, and the results were graphically represented as two‐way hierarchical dendrograms, displayed as heat maps in the case of the VOCs analysis (Josse and Husson 2012). Matlab R2013a (MathWorks, Natick, MA, USA) with PLS Toolbox (Eigenvector Research Inc., Manson, WA, USA) and metaboanalyst 5.0 were employed for multivariate statistical and chemometric analyses.

## 3 Results and Discussion

### 3.1 Chemical Composition

The main quality parameters measured in the study are reported in Table 2.

**TABLE 2:** Quality parameters detected in the different hazelnut samples.

Sample	MC (%)	AT (% oleic acid)	PV (mEqO2/Kg)	TSS (°Brix)	PC g/100 g
Fresh	^a^11.46 ± 3.72	^a^1.08 ± 0.04	^b^0.03 ± 0.01	^a^4.25 ± 0.17	^a^9.43 ± 0.22
In‐Shell	^b^5.23 ± 3.07	^b^0.83 ± 0.04	^b^0.03 ± 0.02	^a^4.24 ± 0.33	^b^6.23 ± 0.52
Shelled	^b^4.53 ± 1.27	^b^0.93 ± 0.05	^b^0.04 ± 0.02	^a^4.32 ± 0.11	^b^6.52 ± 0.33
Selected Shelled	^b^5.07 ± 1.11	^c^0.21 ± 0.01	^b^0.02 ± 0.01	^a^4.39 ± 0.54	^b^6.36 ± 0.27
Low Roasted	—	^c^0.24 ± 0.03	^a^0.10 ± 0.01	^b^2.28 ± 0.11	^c^3.88 ± 0.03
Medium Roasted	—	^c^0.31 ± 0.02	^a^0.11 ± 0.03	^b^2.31 ± 0.15	^c^3.01 ± 0.16
High Roasted	—	^c^0.33 ± 0.05	^a^0.14 ± 0.01	^b^2.13 ± 0.09	^c^2.52 ± 0.15

*Note:* Value is the mean ± sd of three replicates. Different letters within the columns represent statistical significance based on one‐way ANOVA and Tukey's post hoc test (per p ≤ 0.05).

Abbreviations: AT = titratable acidity, MC = moisture content, PC = protein content, PV = peroxide values, TSS = total soluble solids.

MC is a crucial parameter to ensure the safety of raw hazelnuts from a hygienic and sanitary point of view. For this reason, harvested fresh hazelnuts, before being sorted, undergo an initial heat treatment aimed at reducing their basic water content. MC analysis was carried out exclusively on raw hazelnuts, revealing significant changes in water presence following the heat treatment applied. Fresh, unsorted, in‐shell hazelnuts showed an average MC of 11.46%, while in other samples, specifically those related to thermally treated hazelnuts, an average MC of 5.23% for unsorted and in‐shell hazelnuts, 4.53% for unsorted and shelled hazelnuts, and 5.07% for selected shelled hazelnuts was observed (Table 2). MC above 12% makes the product an ideal substrate for pathogenic microorganisms' growth and contributes to a potential decrease in the product shelf life (Kaveh et al. 2018). The product shelf life upon entering a processing company is a very important variable since incoming lots, once selected and calibrated, are generally not immediately destined to be processed or packed, but are stored at controlled temperature and humidity conditions. Therefore, ensuring the preservation of qualitative characteristics for a certain period is extremely important. Consequently, given that water activity is a key parameter characterizing hazelnut quality, it is essential to carry out heat treatment as quickly as possible in the post‐harvest period (Ghirardello et al. 2013). The obtained results align with previous publications addressing the effect of heat treatments on hazelnut moisture content, emphasizing the influence of temperature on these levels (Kaveh et al. 2018; Özdemir and Devres 1999).

The TA varies between 1.08% (in fresh hazelnut) and 0.21% (in selected and shelled hazelnut) (Table 2). The higher acidity content observed in the oil of unselected in‐shell hazelnuts, compared to that of selected hazelnuts, is attributed to the presence of defective nuts in the unselected harvested fruits. Such defects, including rot/mold and insect damage (*Gonocerus acuteangulatus* (Goeze) and *Palomena prasina* (L.)), compromise the quality and safety of the final product. The selection process, therefore, not only ensures the safety of the product by removing foreign bodies of various natures but also allows for the highly selective removal of damaged fruits, consequently reducing the overall acidity level in the selected product compared to the bulk hazelnuts directly harvested from the field (Fontana et al. 2014).

The PV content of raw hazelnuts (namely Fresh, In‐Shell, Shelled, and Selected Shelled) was ≤ 0.04 mEqO_2_/Kg, while it was higher in roasted ones, with a small but progressive increase in the content according to the arising temperature (Table 2). This demonstrates the acceleration of the degradative/oxidative phenomena due to the heat treatments. Hence, it is known that high temperatures, during the roasting process, can increase peroxides in hazelnuts (Alamprese et al. 2009; Shafiei et al. 2020). However, the effect of high temperature on peroxides may vary depending on the roasting method. For instance, several works point out that as the process temperature increases, an increase in peroxide values is observed, as observed in the present study (Amaral et al. 2006; Binello et al. 2018). Other studies found that roasting hazelnuts at 120°C for 40 min by a hot air method resulted in an increase in the oxidative stability (Belviso et al. 2017). On the other hand, roasting hazelnuts through a microwave system did not significantly affect peroxide value (Kalkan et al. 2015).

Roasting hazelnuts at different temperatures can also affect the sugar and protein content (Lamberti et al. 2021) PC and TSS decreased when hazelnuts were subjected to roasting (Table 2). The contents varied as a function of the heat treatment, proportionally decreasing as the temperature increased (Özdemir et al. 2001). Previous studies reported that at higher temperatures, a decrease in protein is observed. This is primarily due to the denaturation of proteins, a process in which the protein structures unfold and lose their functional properties. Additionally, the Maillard reaction and caramelization, which occur during hazelnut roasting, lead to changes in the concentrations of sugars and amino acids (Göncüoğlu Taş and Gökmen 2017). Furthermore, it is important to note that under the three different temperature conditions tested, no statistically significant differences were observed in either protein content or sugar concentrations. This suggests that, within the range of temperatures used (140°C, 150°C, and 160°C), the roasting process did not lead to substantial variations in the protein or sugar levels, indicating a degree of stability in these components despite the changes in roasting conditions.

### 3.2 Spectral Analysis and Prediction of Quality Parameters

**FIGURE 1:**
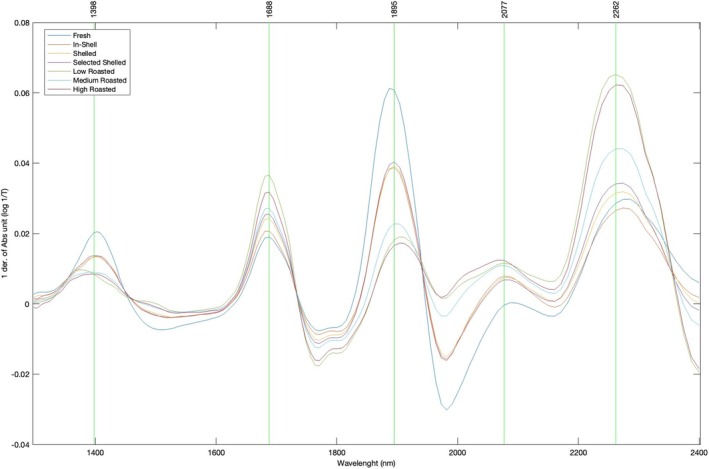
Average spectra in Absorbance (log 1/T), obtained from FT‐NIR acquisitions pre‐treated by applying Savitzky–Golay filtering (1st derivative, smoothing over 25 points), referred to various hazelnut sample groups. Marked peaks with the highest absorbance correspond to the vibrational behavior of resonating functional groups at specific wavelengths.

The chemical analyses were coupled with non‐destructive measurements performed through spectral acquisitions and addressed to chemometric investigations. In Figure 1, the average spectra of hazelnuts referred to the different sample groups are shown. As reported in the literature, several peaks (vibrational centers) of significant relevance are observed (Hirano et al. 1998; Ayvaz et al. 2022). Specifically, in the terminal portion of the spectrum, absorbance bands around 2060 and 2100 nm can be attributed to lipid sources (C‐H and C‐O). Peaks observed at 2100 nm suggest a plausible correlation with protein amide groups (Aykas and Menevseoglu 2021) Resonance centers in the 2200–2260 nm range describe the stretching and deformation combinations of C‐H and C‐H_2_ groups belonging to carbohydrates, and peaks at 1450 and 1920 nm are assignable to the first overtone in stretching of free OH and combination bands of OH related to water, despite the relatively low water content of hazelnuts (Ayvaz et al. 2022; Franco et al. 2006).

**FIGURE 2:**
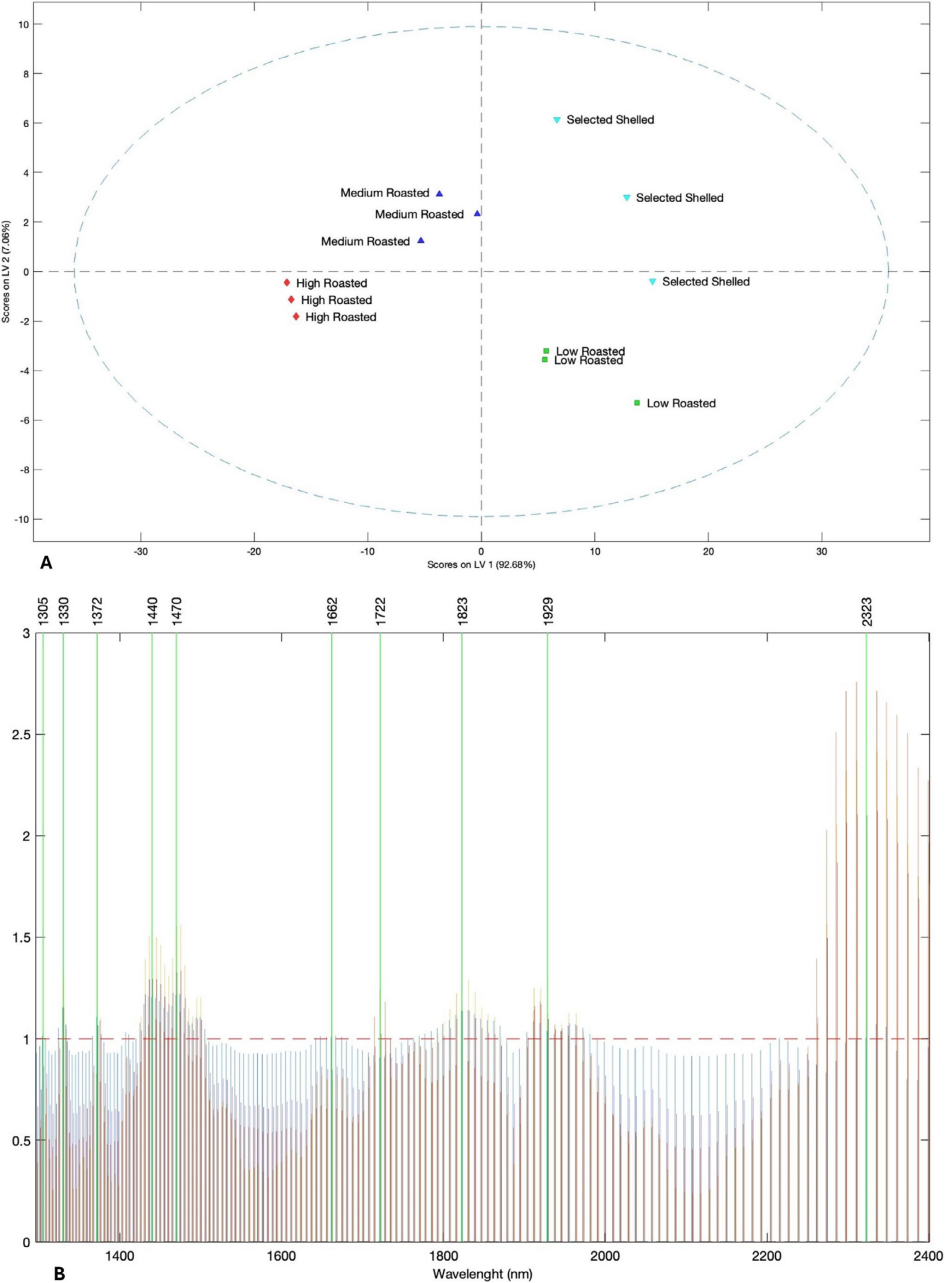
PLS‐DA model computed on autoscaled absorbance FT‐NIR spectra. The employed dataset only includes data from selected raw shelled hazelnuts and roasted hazelnuts at low (140°C), medium (150°C), and high (160°C) roasting conditions. (Panel A) Scoreplot (LV1 vs. LV2) including explained % of variance reported in the axis titles. (Panel B) VIP scores describing the spectral portions most influencing the segregation of the samples in the PLS‐DA modeling.

Raw spectral data were projected into a PLS‐DA model to verify the aptitude of the NIR detection to discriminate and group the samples based on the processing treatment. A preliminary PLS‐DA model was developed including all sample classes spectrally measured, also considering fresh hazelnut deshelled having a relatively high‐water content (data not shown). However, as expected, the obtained segregation was significantly affected by the wavelengths associated with the vibrational response of water molecules. In order to address the PLS‐DA discrimination exclusively towards the impact of different processing conditions on hazelnut samples, the final model was developed using a dataset only including the groups of roasted and fresh deshelled hazelnuts, having an average water content of 5.07%, to be destined for the roasting process. Figure 2a shows the resulting score plot of the PLS‐DA model, illustrating the projection of the scores onto the first two latent variables (LV1 vs. LV2). The model explains 99.74% of the total variability in the data, with LV1 contribution at 92.98%, and LV2 at 7.06%. The graph clearly demonstrates a division of the groups across the four quadrants: scores deriving from the NIR spectra related to raw hazelnuts and low‐temperature roasted hazelnuts are located in the 2nd and 3rd quadrants, respectively, while those referred to the medium‐ and high‐temperature roasted hazelnuts are located in 1st and 4th quadrants. It is noteworthy that the sample segregation based on LV1 closely mirrors the segregation observed in the PCA analysis computed on the destructive data (Figure S1). In both analyses, the selected raw hazelnuts and low‐temperature roasted hazelnuts are positioned in a diametrically opposite quadrants when compared to the medium‐ and high‐temperature roasted hazelnuts. This shows that the raw spectral information accurately reflects the differentiation of samples based on destructive measurements, by supporting the observation that NIR spectral response is significantly affected by the vibrational response of the molecules findable in the inspected matrix. The model robustness is further confirmed by Table 3, which reports the confusion matrices and tables in calibration and cross‐validation. These matrices show how all samples (*n* = 12) were correctly classified into their respective groups, both in calibration and prediction computation, also demonstrating the model's reliability in successfully distinguishing among the different roasting conditions of hazelnuts. On the other hand, Figure 2b shows the variable importance projection (VIP) scores associated with the PLS‐DA model, highlighting four main significant spectral regions that affect the score segregations and classifications. The regions between 1420–1500 nm and 1900–1950 nm are associated with the spectral response of the water molecules. These wavelength portions are assigned to the first overtone of the symmetric and asymmetric OH stretching and/or combination bands (1450 nm) and to the combination of the OH stretching band and the OH bending band (1920–1950 nm), respectively (Franco et al. 2006; Shenk and Westerhaus 1996; Nicolaï et al. 2007). The spectral region included between 1750 and 1850 nm corresponds to the first and second overtones of the CH stretching vibrations in CH_3_, CH_2_, and CH=CH groups, which can be associated with the structural presence of fatty acids (Shenk and Westerhaus 1996). Finally, the region between 2280 and 2400 nm, which can be associated with the combination band of the stretching and deformation vibrations of the ‐CH_2_ groups attributable to lipids (Osborne et al. 1993; Williams and Norris 1987), shows the most significant impact on the PLS‐DA results.

**TABLE 3:** Matrices and model confusion tables in calibration and prediction for the PLS‐DA shown in Figure 2.

*Confusion Matrix* (*Calibration*)								
**Class**	**TPR**	**FPR**	**TNR**	**FNR**	***N***	**Err**	***p***	***F*1**
Selected Shelled	1.00	0.00	1	0.00	3	0.00	1.00	1.00
Low Roasted	1.00	0.00	1	0.00	3	0.00	1.00	1.00
Medium Roasted	1.00	0.00	1	0.00	3	0.00	1.00	1.00
High Roasted	1.00	0.00	1	0.00	3	0.00	1.00	1.00
*Confusion Table* (*Calibration*)								
**Predicted as:**	**Selected shelled**		**Low roasted**		**Medium roasted**		**High roasted**	
Selected Shelled	3		0		0		0	
Low Roasted	0		3		0		0	
Medium Roasted	0		0		3		0	
High Roasted	0		0		0		3	
*Confusion Matrix* (*CV*)								
**Class**	**TPR**	**FPR**	**TNR**	**FNR**	***N***	**Err**	***p***	***F*1**
Selected Shelled	1.00	0.00	1	0.00	3	0.00	1.00	1.00
Low Roasted	1.00	0.00	1	0.00	3	0.00	1.00	1.00
Medium Roasted	1.00	0.00	1	0.00	3	0.00	1.00	1.00
High Roasted	1.00	0.00	1	0.00	3	0.00	1.00	1.00
*Confusion Table* (*CV*)								
**Predicted as:**	**Selected shelled**		**Low roasted**		**Medium roasted**		**High roasted**	
Selected Shelled	3		0		0		0	
Low Roasted	0		3		0		0	
Medium Roasted	0		0		3		0	
High Roasted	0		0		0		3	

The NIR acquisitions were also used to explore the correlations between NIR characteristics and crucial quality indicators such as MC, TA, PV, PC, and TSS. Table S1 provides information on the sample sets of destructive data used for modeling correlations (dependent variable Y), including sample size, mean, minimum, maximum, and standard deviation.

Regarding the PLS model for MC prediction, raw spectra were transformed into absorbance and, in turn, treated with various chemometric filters (namely, Savitzky–Golay filter for 1st and 2nd derivatives at 15 and 25 smoothing points, respectively, and SNV—standard normal variate). Subsequently, they underwent computational regression for PLS modeling. The model performances are reported in Table S2. The best performance was achieved by employing spectra transformed from original transmittance values to absorbance (log1/T) and pre‐treated with a Savitzky–Golay filter for 1st derivative at 15 smoothing points. The model exhibits a correlation of 0.918 and 0.870 in prediction and calibration, respectively, with errors of 0.55% and 0.69% (Figure S2). The good predictive performance of the model is also observed in the score plot, showing clear segregation between raw hazelnuts (with higher moisture content) and hazelnuts subjected to pre‐drying treatment (data not shown). However, in terms of the model predictive reliability, the distribution of destructive data negatively affects the predictive regression result, as evidenced by the large ‘prediction gap’ and the considerable number of latent variables (7 LVs) required to minimize predictive error. In this regard, when evaluating the efficiency and statistical robustness of the PLS models, it is essential to consider that a small reduction in the goodness of the correlation obtained with a model computed from a simple spectral transformation from transmittance to absorbance (0.871 and 0.859, respectively, for R^2^cal and R^2^cv) is more than compensated by a significant reduction in the number of latent variables (5) needed to minimize predictive error and relative values of estimation errors (0.641% and 0.725%, respectively, in calibration and prediction).

Bellincontro et al. (2005), working with a non‐interferometric spectrophotometer operating in the wavelength range of 1100–2500 nm and acquiring data from 50 whole hazelnuts, obtained regression models with correlation values referred to % of MC that are consistent with results performed here (0.895 and 0.880 for R^2^cal and R^2^cv).

The good statistical variability is reflected in the RPD (relative percent deviation) index reported in Table S2, indicating the performance of predictive models compared with the standard deviation (SD) of destructive data and mean prediction error (RMSECV) (Fearn 2002; Nicolaï et al. 2007). The RPD obtained for the selected model is 3.49, a value above the threshold of good predictive performance, which some authors have estimated to be equal to 3 (Nicolaï et al. 2007; Williams and Sobering 1996; Fearn 2002).

PLS model associated with TA prediction shows a correlation of 0.753 and 0.537 in calibration and prediction, respectively, with 5 LVs minimizing the error (RMSEC and RMSECV) at 0.16% and 0.23% (Table S3). As for the model developed for the evaluation of PV, correlations are significantly more satisfactory compared to the previous model, with correlations of 0.919 (R^2^cal) and 0,809 (R^2^cv). With respect to the model built for PC, high determination indices of 0.951 (Cal) and 0.902 (CV) were obtained, with RMSEC and RMSECV of 0.52 and 0.74, respectively, minimized by 5 LVs. Finally, regarding the model performed for TSS, correlations of 0.933 (R^2^ Cal) and 0.786 (R^2^ CV) were obtained.

In general, the computed models demonstrate promising potential—or notable limitations depending on the specific parameters tested—in correlating destructive and non‐destructive data. Specifically, strong confidence was observed in the prediction of parameters such as PV, PC, and TSS. However, lower efficiency was noted for parameters such as TA. Beyond the modeling applied to the percentage of moisture content (%MC), which was analytically determined on individual whole hazelnuts and developed using a sufficiently rich and variable dataset, the other models showed clear limitations due to the restricted amount of processed data. These models require further refinement and the inclusion of more comprehensive datasets to achieve more robust and reliable validations.

### 3.3 Aromatic Characterization: VOCs Detection and E‐Nose Profile

The roasting process significantly affects the aromatic profile of hazelnuts by influencing the physical–chemical properties and the origin of released volatile compounds. Different pre‐drying and roasting conditions can result in aroma and flavor modifications (Binello et al. 2018; Marzocchi et al. 2017).

**FIGURE 3:**
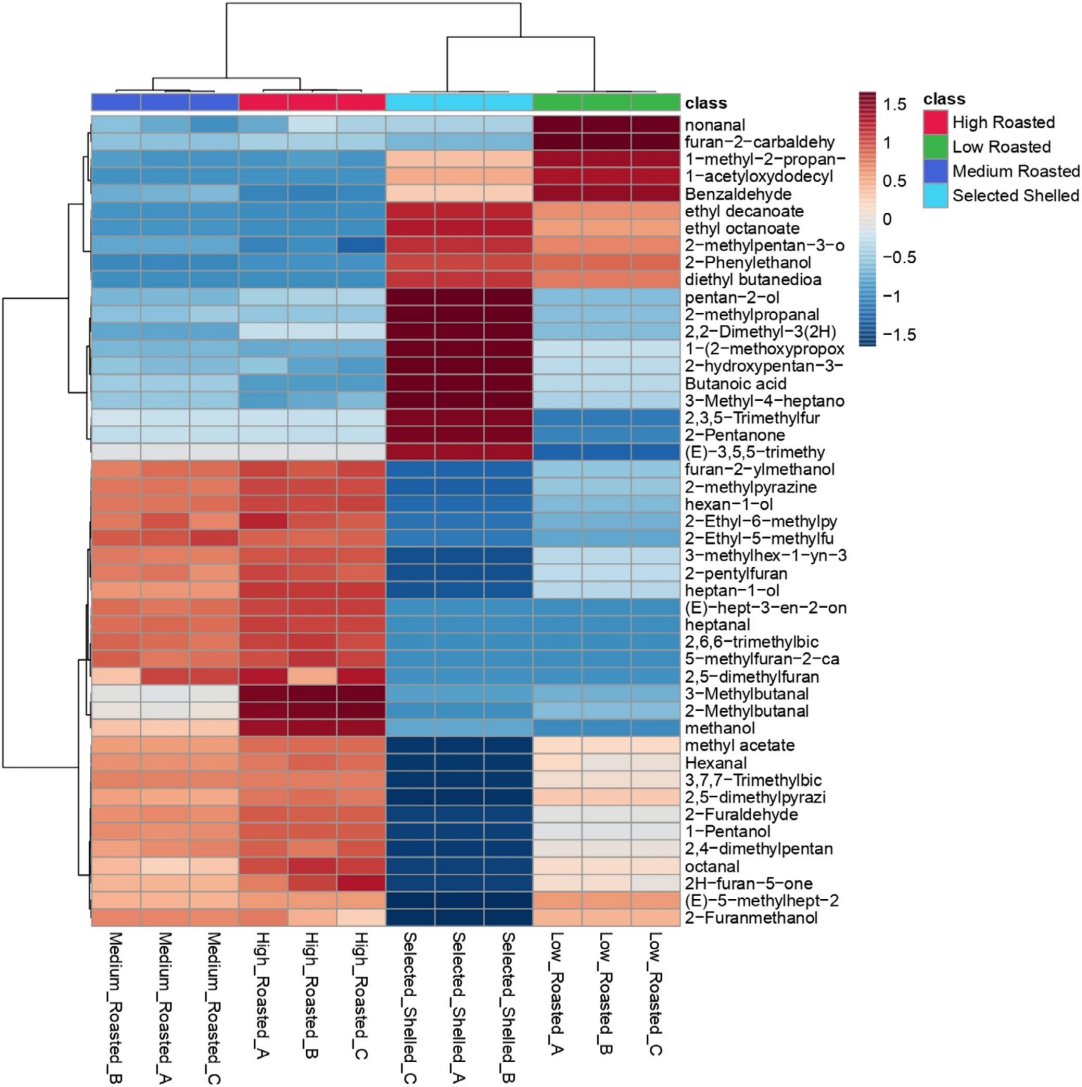
Heat‐Map representing the results obtained from cluster analysis (CA) performed on the GC–MS data detected on hazelnut samples. CA was computed by applying the Ward's method under PCA calculation.

Through the SPME GC–MS technique, a total of 47 volatile compounds were identified in the different hazelnut samples, differently findable with respect to sample condition (Table S4). A cluster analysis, performed using Ward's method on VOC data, was employed to provide a deeper understanding of the influence of individual volatiles on the observed clustering of hazelnut classes. Samples segregate into two main clusters, each affected by distinct aromatic patterns (Figure 3): a first cluster attributed to unroasted and low roasted hazelnuts and a second one including medium and high roasted hazelnuts. Hierarchical distance is lower between medium and high roasted classes within the same cluster than that observed between unroasted and low roasted ones in the other cluster. Hazelnuts subjected to roasting treatments (low, medium, and high roasting) are characterized by a wide range of furfural compounds, which are known to be a natural consequence of heat treatments. After roasting treatments, a significant increase in furfural, 2,4‐dimethylpentan‐3‐ol, (E)‐5‐methylhept‐2‐en‐4‐one, 2H‐furan‐5‐one, 2‐Furanmethanol, methyl acetate, 2‐methylpyrazine, furan‐2‐carbaldehyde, and hexanal was observed. Medium and high roasted hazelnuts also showed an increase in several specific compounds not found in low roasted ones, including 2,5‐dimethylfuran, 5‐methylfuran‐2‐carbaldehyde, 2,6,6‐trimethylbicyclo[3.1.1]hept‐2‐ene, heptanal, methanol, 2‐and 3‐methylbutanal, 2‐Furylmethanol, and 2‐pentylfuran. Among these compounds, heptanal and 2‐pentylfuran are significantly relevant from an olfactory perspective. Heptanal, an aldehyde with a seven‐carbon chain, is characterized by its fatty odor profile, while 2‐pentylfuran, a furan derivative with a five‐carbon chain and a pentyl group, is distinguished by its buttery aroma, adding a creamy, smooth quality to the overall scent profile (Kiefl and Schieberle 2013). Moreover, 2‐methylbutane and 3‐methylbutane, produced through the Strecker degradation of amino acids during the Maillard reaction, are particularly appealing to consumers due to their association with minty aroma notes. Furthermore, while roasting treatments, by triggering the Maillard reaction, promote a decrease in some compositional parameters such as TSS and PC, they also determine a significant increase in several VOCs that are particularly appreciated from a sensory point of view (Kiefl and Schieberle 2013; Stilo et al. 2021; Cialiè Rosso et al. 2018; Alasalvar et al. 2003; Nicolotti et al. 2013; Erten and Cadwallader 2017).

Raw hazelnuts present a distinct flavor profile compared to medium and high roasted classes, and just partially similar to those included in the low roasting class. Some compounds, namely butanoic acid and 2‐pentanone, are exclusively characteristic of raw hazelnuts, and they are commonly associated with sweet and fruity flavors (Stilo et al. 2021). These compounds significantly affect the segregation of grouped raw hazelnuts. After the roasting treatments, the same VOCs and volatile categories to which they refer (mainly alcohols and ketones) are lost.

Low roasted and raw hazelnuts are characterized by a pool of compounds detected in both classes, mainly volatile fatty acids and related ethyl esters (in detail, ethyl decanoate, ethyl octanoate, 2‐methylpentan‐3‐ol, 2‐Phenylethanol, diethyl butanedioate). Among these VOCs, ethyl octanoate is responsible for fruity and waxy nuances, while 3‐methyl‐2‐pentanol is responsible for green and fruity aromas (Stilo et al. 2021; Cialiè Rosso et al. 2018). Additionally, in low‐roasted hazelnuts, nonanal, a potent odorant that imparts fruity notes, was detected (Kiefl and Schieberle 2013). Interestingly, the cluster grouping of raw and low‐roasted hazelnuts reveals a common thread characterized by similar descriptors originating from different molecules. GC–MS is an analytical method highly performant in detecting and identifying different and specific volatiles. On the other hand, an E‐nose is based on a sensor array that mimics the human olfactory sense. E‐nose and GC–MS are both valuable tools for food analysis, but they differ in their working principles and applications. E‐nose offers rapid and non‐destructive analysis, wide applicability, and cost‐effectiveness, while GC–MS provides high sensitivity, specificity, and quantitative analysis capabilities. E‐nose detection is based on the acquisition of a specific aromatic pattern, which is characteristic of a single product, so it is an analytical technique specifically addressed to make comparisons, being coupled with chemometrics (Modesti, Taglieri, et al. 2022).

**FIGURE 4:**
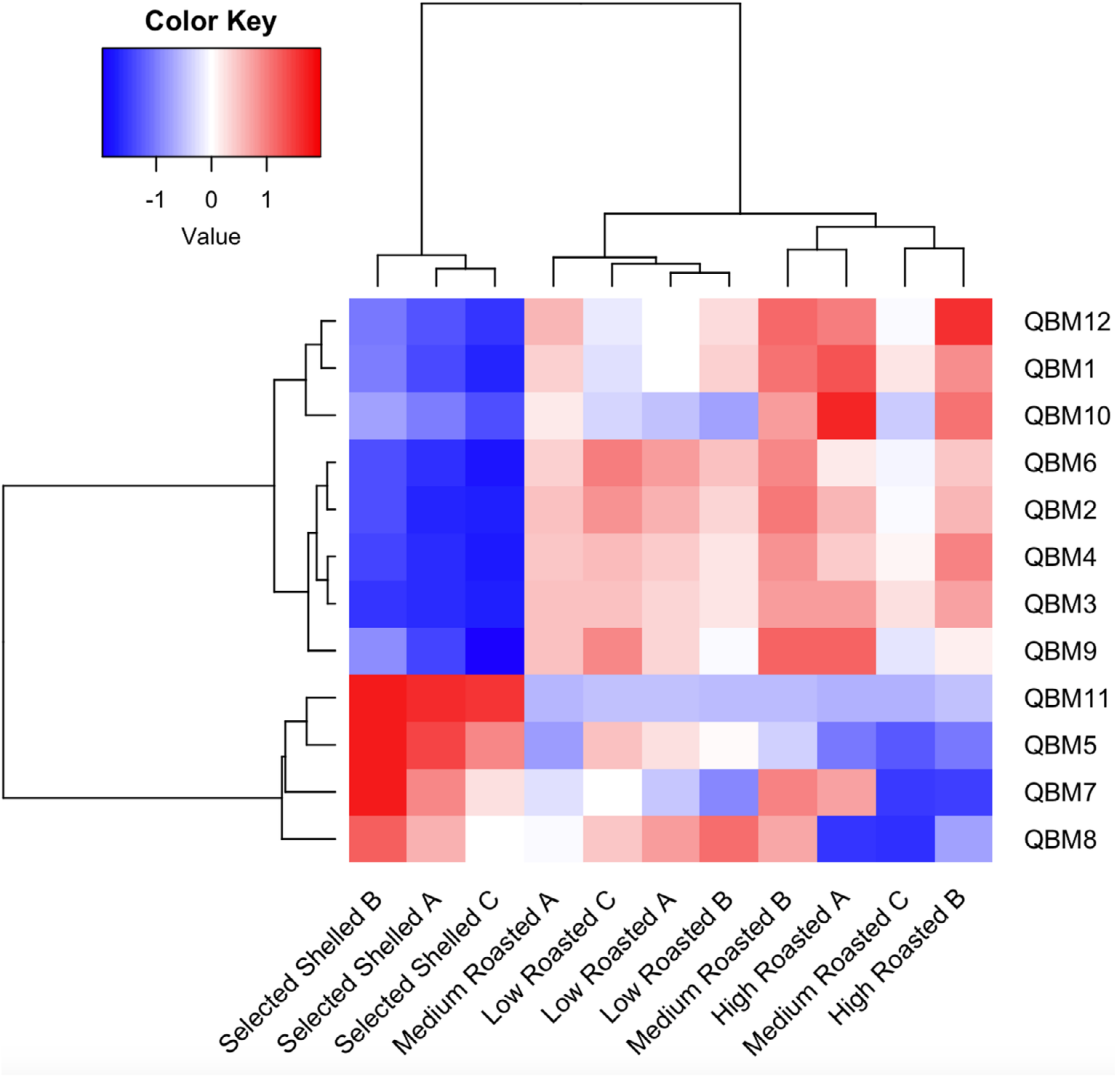
Hierarchical dendrograms resulting from the two‐way cluster analysis (CA) performed on the E‐nose data detected on hazelnut samples. CA was computed by applying the Ward's method under PCA calculation.

Volatiles released by the hazelnut have also been analyzed through an E‐nose equipped with twelve QMB sensors, each functionalized with different metalloporphyrins. In Figure 4, the dendrograms resulting from the two‐way CA computed on E‐nose data are shown. The segregation of the hazelnut samples is well described in combination with the clustering effect due to the loading (the QMBs) influence. The obtained results describe a quite good clustering effect: the class of raw hazelnuts (selected and shelled) segregates differently from roasted ones. The segregation of roasted hazelnuts is further divided into two: one with the low‐temperature roasted hazelnuts and the other with medium and high temperature roasted samples. QMB 11 (phosphorus complex), QMBs 5 and 7 (cobalt complex), and QMB 8 (ruthenium complex) are the E‐nose sensors most characterizing the cluster associated with raw hazelnuts. Other authors working with the same E‐nose device on the volatiles released by *Aspergillus* spp. found a significant correlation between the responses of QMBs cobalt‐functionalized and the presence of acetic acid and ethanol (Capuano et al. 2020). Even though acetic acid and ethanol were not detected in tested hazelnuts, it is plausible that structurally related compounds influenced the same microbalances, thus generating similar signals. The other QMBs (10, 1 and 12) were found to be more sensitive to the aromatic pattern released by roasted hazelnuts. Specifically, QMB 10 (copper complex) has been found to interact with polar volatile components, which are well known to increase during the roasting process (Capuano et al. 2020). As previously described, the volatile compounds characterizing roasted hazelnuts at 150°C and 160°C are mostly polar. This polarity is attributed to the presence of functional groups such as hydroxyl (‐OH), carbonyl (C=O), and carboxyl (‐COOH). Among the identified polar compounds, alcohols, aldehydes, and organic acids can be easily mentioned, and they are known to contribute to the distinctive aromatic notes and sensory profile complexity of roasted hazelnuts.

The hazelnut class separation based on E‐nose is only partially comparable with that based on VOC determination carried out by GC–MS analysis. In fact, in this last evaluation, raw hazelnuts were closer to the low roasted ones, while medium and strong roasted segregated in a distinct cluster. Discrimination of hazelnut classes based on VOCs only partially reflects human olfactory perception which, as reported before, is instead the aptitude most close to the E‐nose working operation. By GC–MS detection, volatiles are evaluated for their presence and, possibly, for their concentration, as a consequence of the equilibrium between the source matrix and the headspace. Because of the possible effects of masking, overlapping, and synergistic interactions of VOCs, the concentration of volatiles is not always synonymous with the odor perception. On the other hand, the E‐nose succeeds in intercepting the overall aroma pattern and allows a more accurate discrimination of roast classes based on the temperature conditions of processing. The E‐nose device showed to be sensitive to the aroma released by the nuts, discriminating the temperature impact on the roasting treatment, a factor that significantly affects the aromatic complexity of hazelnuts (Cialiè Rosso et al. 2018). Therefore, the E‐nose represents a valuable tool for non‐destructive aroma‐based discrimination of hazelnuts. There are limited studies in the literature on the application of the E‐nose for hazelnut analysis. Alasalvar et al. (2012), in their work on Turkish hazelnuts, demonstrated the aptitude of an E‐nose MOS sensor‐based system to discriminate among different varieties of raw and roasted hazelnuts.

Investigations conducted by Binello et al. (2018) on PGI Piedmont hazelnuts, using an E‐nose coupled to chemometric modeling, demonstrated the device's performance in differentiating between two roasting methods, performed by infrared and hot air processing. The experimental trial explored a range of time and temperature combinations (45 min at 135°C and 27 min at 195°C for hot air roasting, and 40 min at 135°C and 20 min at 195°C for infrared treatment). The authors demonstrated clear differences in relation to the roasting method, and slight variations between temperature conditions and time even though there was a large gap between 135°C and 195°C. The approach followed in our experimental trial, a more limited reaching‐up of roasting temperature (10°C, from 140°C to 160°C) with a standardized time of processing (24 min), induced evident changes in the aromatic profiles of hazelnuts well described by the VOC profiles and well detected via E‐nose evaluation.

## 4 Conclusions

The roasting process of hazelnut is, in the food industry, a key procedure highly affecting the quality characteristics of the product aimed at being processed mainly for successive food preparations. The processing conditions, particularly the roasting temperatures, have a significant impact on volatile compound release, as well as on the aromatic profiles of hazelnuts, and the monitoring of their behaviors can have a strategic role in the final quality of the dried fruits. Traditional techniques for the determination of the main quality attributes of hazelnuts are time and labor‐consuming and often, from a farm perspective, they are misaligned to the rapid control of incoming and outgoing fruits and, particularly, to the efficient monitoring of the fruit processing. The recourse to non‐destructive analytical techniques sensor‐based, combined with chemometric computing, can offer an advantage in overcoming this limitation. In the present study, FT‐NIR spectroscopy revealed its aptitude to be employed for a regressive prediction of hazelnut moisture content, based on the well‐known vibrational response due to physical–chemical interaction between spectra and molecules. Three roasting temperatures, under operative industrial conditions, have been tested on hazelnuts, and allowed us to observe significant differences in terms of fruit quality and in relation to its monitoring. Analysis of VOCs detected by GC–MS inspection provided well‐defined and separated aromatic profiles, proving to distinguish fresh unroasted hazelnuts from roasted ones, with an evident separation between hazelnuts processed at 140°C and at 150°C–160°C, and a greater similarity between fresh and lower temperature‐treated fruits. A very similar response in terms of sample discrimination patterns has been obtained by evaluating the hazelnut groups via FT‐NIR spectroscopy and E‐nose detection, combined with multivariate chemometric approaches. This demonstrated the feasibility of non‐destructive analyses in predicting the quality of hazelnuts based on the internal attribute modification due to the roasting treatments, thus proposing themselves as potential high‐performance tools for process monitoring.

## Author Contributions

**Riccardo Riggi:** data curation (equal), formal analysis (equal), writing – original draft (equal), writing – review and editing (equal). **Margherita Modesti:** conceptualization (equal), data curation (equal), investigation (equal), supervision (equal), writing – original draft (equal), writing – review and editing (equal). **Gianmarco Alfieri:** formal analysis (equal), methodology (equal). **Giuseppe Esposito:** conceptualization (equal), resources (equal), supervision (equal). **Paolo Cucchiara:** conceptualization (equal), resources (equal), supervision (equal). **Serena Ferri:** formal analysis (equal), methodology (equal), writing – review and editing (equal). **Fabio Mencarelli:** conceptualization (equal), supervision (equal), writing – review and editing (equal). **Andrea Bellincontro:** conceptualization (equal), data curation (equal), supervision (equal), writing – original draft (equal), writing – review and editing (equal).

## Conflicts of Interest

The authors declare no conflicts of interest.

## Supporting information


Data S1.

## Data Availability

All data generated and analyzed during this study are included in the main article and its Supporting Information S1.
