# Supplementary material for: Integration of Destructive and Non‐Destructive Analytical Determinations for Evaluating Quality of Fresh and Roasted Hazelnuts Subjected to Different Processing Temperatures
Source: Food Sci Nutr. 2025 Mar 12;13(3):e70095. doi: 10.1002/fsn3.70095 (PMC11904111; doi:10.1002/fsn3.70095)
Supplement: Supplementary file 1 — Data S1. [file FSN3-13-e70095-s001.docx]

**Supplementary materials**

**Table & Figure captions**

**Table S1** Data matrix providing information on the sample sets of destructive analyses used for regressive modelings.

**Table S2** Regressive performances obtained opposing FT-NIR spectra (independent variable), subjected to different chemometric pre-treatments, to the hazelnut moisture content (MC, dependent variable) in PLS modeling. Reported indexes include: R² (Coefficient of Determination) in calibration (R²_cal) and cross-validation (R²_cv), root mean square error of calibration (RMSEC), root mean square error of cross-validation (RMSECV), the number of latent variables (LVs), and the Ratio of Performance to Deviation (RPD).

**Table S3** Regressive performances obtained opposing FT-NIR spectra (X-block, independent variable) to different chemical attributes of hazelnut (Y-block, dependent variables); in detail, total acidity (AT), peroxide index (PV), protein content (PC), and total soluble solids (TSS). Reported indexes include: R² (Coefficient of Determination) in calibration (R²_cal) and cross-validation (R²_cv), root mean square error of calibration (RMSEC), root mean square error of cross-validation (RMSECV), the number of latent variables (LVs), and the Ratio of Performance to Deviation (RPD).

**Table S4** Relative percentage of volatile compounds (VOCs) detected in hazelnut samples by GC-MS measurement. The value is the mean ± sd of three replicates. Different letters within the columns represent statistical significance based on one-way ANOVA and Tuckey's post hoc test (with p ≤ 0.05).

**Figure S1** Scoreplot of principal component analysis (PCA), computed on destructive data.

**Figure S2** Scatterplot relative to partial least squares (PLS) regressive model for moisture content computed on detected FT-NIR spectra.

Table S1 Data matrix providing information on the sample sets of destructive analyses used for regressive modeling.

|  | **MC (%)** |
| --- | --- |
| **n** | 221 |
| **Mean** | 5.06 |
| **min** | 2.25 |
| **Max** | 12.39 |
| **Std. dev. (±)** | 1.93 |

| ID | **TA** (%) | **PV** (meqO_2_/Kg) | **PC** (g/100g) | **TSS** (Brix °) |
| --- | --- | --- | --- | --- |
| Fresh | 1.10 | 0.03 | 9.88 | 4.11 |
| Fresh | 1.09 | 0.04 | 9.74 | 4.20 |
| Fresh | 1.03 | 0.02 | 10.18 | 4.44 |
| In-Shell | 0.82 | 0.05 | 6.57 | 4.03 |
| In-Shell | 0.88 | 0.01 | 6.47 | 4.07 |
| In-Shell | 0.79 | 0.02 | 5.63 | 4.62 |
| Shelled | 0.99 | 0.06 | 6.14 | 4.44 |
| Shelled | 0.92 | 0.05 | 6.78 | 4.21 |
| Shelled | 0.89 | 0.02 | 6.64 | 4.33 |
| Selected Shelled | 0.22 | 0.01 | 6.20 | 3.95 |
| Selected Shelled | 0.22 | 0.03 | 6.68 | 5.00 |
| Selected Shelled | 0.21 | 0.02 | 6.20 | 4.23 |
| Low Roasted | 0.26 | 0.10 | 3.84 | 2.41 |
| Low Roasted | 0.25 | 0.12 | 3.88 | 2.22 |
| Low Roasted | 0.21 | 0.11 | 3.91 | 2.21 |
| Medium Roasted | 0.29 | 0.12 | 2.83 | 2.14 |
| Medium Roasted | 0.32 | 0.11 | 3.07 | 2.35 |
| Medium Roasted | 0.32 | 0.12 | 3.14 | 2.43 |
| High Roasted | 0.37 | 0.14 | 2.69 | 2.13 |
| High Roasted | 0.28 | 0.11 | 2.39 | 2.04 |
| High Roasted | 0.33 | 0.16 | 2.48 | 2.21 |
| **n** | 21 | 21 | 21 | 21 |
| **Mean** | 0.53 | 0.07 | 5.27 | 3.38 |
| **Min** | 0.21 | 0.01 | 2.39 | 2.04 |
| **Max** | 1.10 | 0.16 | 10.18 | 5.00 |
| **Std. dev. (±)** | 0.35 | 0.05 | 2.45 | 1.07 |

Table S2. Regressive performances obtained opposing FT-NIR spectra (independent variable), subjected to different chemometric pre-treatments, to the hazelnut moisture content (MC, dependent variable) in PLS modeling. Reported indexes include: R² (Coefficient of Determination) in calibration (R²_cal) and cross-validation (R²_cv), root mean square error of calibration (RMSEC), root mean square error of cross-validation (RMSECV), the number of latent variables (LVs), and the Ratio of Performance to Deviation (RPD).

| Pre-treatment | **R^2^cal** | **R^2^cv** | **RMSEC** | **RMSECV** | **LV** | **RPD** |
| --- | --- | --- | --- | --- | --- | --- |
| - | 0.822 | 0.803 | 0.8146 | 0.8577 | 5 | 2.37 |
| **ABS** | 0.871 | 0.859 | 0.6412 | 0.72495 | 5 | 3.01 |
| **SG derivatives 1^** | 0.918 | 0.870 | 0.55288 | 0.69646 | 7 | 3.49 |
| **SG derivatives 2^** | 0.915 | 0.864 | 0.56424 | 0.71374 | 14 | 3.42 |
| **SNC** | 0.914 | 0.808 | 0.5651 | 0.8607 | 16 | 3.41 |

Table S3 Regressive performances obtained opposing FT-NIR spectra (X-block, independent variable) to different chemical attributes of hazelnut (Y-block, dependent variables); in detail, total acidity (AT), peroxide index (PV), protein content (PC), and total soluble solids (TSS). Reported indexes include: R² (Coefficient of Determination) in calibration (R²_cal) and cross-validation (R²_cv), root mean square error of calibration (RMSEC), root mean square error of cross-validation (RMSECV), the number of latent variables (LVs), and the Ratio of Performance to Deviation (RPD).

| analytical parameters | **R^2^ cal** | **R^2^ cv** | **RMSEC** | **RMSECV** | **LV** | **RPD** |
| --- | --- | --- | --- | --- | --- | --- |
| **AT** (% oleic acid) | 0.754 | 0.537 | 0.16 | 0.23 | 5 | 1.47 |
| **PV** (meqO_2_/Kg) | 0.919 | 0.809 | 0.01 | 0.02 | 5 | 2.32 |
| **PC** (g/100g) | 0.951 | 0.902 | 0.52 | 0.74 | 5 | 1.43 |
| **TSS** (Brix °) | 0.933 | 0.786 | 0.27 | 0.48 | 5 | 5.06 |

Table S4 Relative percentage of volatile compounds (VOCs) detected in hazelnut samples by GC-MS measurement. The value is the mean ± sd of three replicates. Different letters within the columns represent statistical significance based on one-way ANOVA and Tuckey's post hoc test (with p ≤ 0.05).

| Class | Fresh | In-Shell | Shelled | Selected Shelled | Low Roasted | Medium Roasted | High Roasted |
| --- | --- | --- | --- | --- | --- | --- | --- |
| Methanol | 8.46 ± 0.55 | 10.51 ± 0.25 | 10.7 ± 0.12 | 10.95 ± 0.09 | 9.32 ± 0.19 | 19.82 ± 0.21 | 28.82 ± 0.22 |
| 2-Methylbutanal | 1.67 ± 0.06 | 0.16 ± 0.01 | 0.22 ± 0.02 | 0.31 ± 0.04 | 2.33 ± 0.12 | 5.83 ± 0.23 | 14.83 ± 0.23 |
| 3-Methylbutanal | 0.65 ± 0.04 | 0.07 ± 0.01 | 0.17± 0.01 | 0.31 ± 0.02 | 0.99± 0.06 | 4.49 ± 0.14 | 13.49 ± 0.32 |
| 2-Pentanone | 1.78 ± 0.10 | 10.77± 0.13 | 11.50 ± 0.11 | 12.53 ± 0.34 | 3.04 ± 0.12 | 4.49 ± 0.34 | 3.49 ± 0.25 |
| 2,2-Dimethyl-3 (2H)-furanone | 0.06± 0.00 | 0.27± 0.01 | 0.34± 0.01 | 0.43 ± 0.01 | 0.27 ± 0.01 | 0.26 ± 0.01 | 0.30 ± 0.02 |
| 3,7,7 Trimethylbicyclo [4.1.0]hept-3-ene | 3.15± 2.29 | 0.08± 0.02 | 0.05± 0.00 | 0.02 ± 0.00 | 0.65 ± 0.01 | 0.89 ± 0.01 | 0.90 ± 0.01 |
| 2-Ethyl-5-methylfuran | 0.00± 0.00 | 0.51± 0.06 | 0.30± 0.02 | 0.00 ± 0.00 | 0.55 ± 0.02 | 3.31 ± 0.13 | 3.14 ± 0.11 |
| 2,3,5-Trimethylfuran | 0.49± 0.02 | 0.54± 0.2 | 0.92± 0.03 | 1.47 ± 0.07 | 0.09 ± 0.01 | 0.56 ± 0.02 | 0.54 ± 0.01 |
| furan-2-carbaldehyde | 0.43± 0.06 | 0.51± 0.04 | 0.60± 0.01 | 0.72 ± 0.02 | 2.38 ± 0.12 | 0.79 ± 0.07 | 0.88 ± 0.04 |
| 2-methylpropanal | 0.23± 0.02 | 4.82± 0.05 | 5.25± 0.13 | 5.86 ± 0.08 | 1.09 ± 0.03 | 1.19 ± 0.12 | 1.23 ± 0.10 |
| Hexanal | 1.42± 0.08 | 1.82± 0.09 | 1.25± 0.08 | 0.43 ± 0.01 | 3.36 ± 0.16 | 4.37 ± 0.01 | 4.70 ± 0.10 |
| pentan-2-ol | 3.29± 2.42 | 10.31± 0.43 | 11.45± 0.22 | 0.13 ± 0.01 | 0.03 ± 0.00 | 0.03 ± 0.00 | 0.04 ± 0.00 |
| 3-Methyl-4-heptanone | 1.09± 0.06 | 1.74± 0.02 | 2.10± 0.11 | 2.61 ± 0.01 | 0.79 ± 0.01 | 0.67 ± 0.02 | 0.47 ± 0.03 |
| (E)-3,5,5-trimethylhex-2-ene | 6.39± 0.38 | 2.95± 0.11 | 4.68± 0.12 | 7.16 ± 0.06 | 2.62 ± 0.11 | 4.67 ± 0.04 | 4.67 ± 0.16 |
| 2-Furanmethanol | 0.02± 0.00 | 0.08± 0.02 | 0.17± 0.01 | 0.29 ± 0.01 | 2.28 ± 0.09 | 2.54 ± 0.04 | 2.33 ± 0.25 |
| 2H-furan-5-one | 0.00± 0.00 | 0.03± 0.00 | 0.02± 0.00 | 0.00 ± 0.00 | 0.19 ± 0.01 | 0.22 ± 0.01 | 0.28 ± 0.03 |
| 2-pentylfuran | 0.02± 0.00 | 0.13± 0.02 | 0.12± 0.01 | 0.10 ± 0.01 | 0.54 ± 0.02 | 0.94 ± 0.03 | 1.22 ± 0.08 |
| 1-Pentanol | 0.30± 0.01 | 1.48± 0.06 | 0.93± 0.02 | 0.15 ± 0.00 | 0.79 ± 0.01 | 1.12 ± 0.01 | 1.23 ± 0.01 |
| Butanoic acid | 0.73± 0.01 | 0.71± 0.03 | 0.81± 0.03 | 0.95 ± 0.02 | 0.44 ± 0.01 | 0.40 ± 0.02 | 0.30 ± 0.01 |
| 2-methylpyrazine | 0.07± 0.00 | 0.00± 0.00 | 0.00± 0.00 | 0.00 ± 0.00 | 0.76 ± 0.01 | 1.74 ± 0.02 | 1.92 ± 0.01 |
| 3-methylhex-1-yn-3-ol | 0.04± 0.00 | 0.05± 0.00 | 0.08± 0.00 | 0.12 ± 0.01 | 3.41 ± 0.01 | 6.58 ± 0.11 | 7.24 ± 0.09 |
| octanal | 0.00± 0.00 | 0.30± 0.02 | 0.19± 0.01 | 0.03 ± 0.00 | 0.22 ± 0.01 | 0.24 ± 0.00 | 0.33 ± 0.02 |
| (E)-5-methylhept-2- en-4-one | 0.25± 0.01 | 0.30± 0.03 | 0.34± 0.01 | 0.38 ± 0.02 | 12.00 ± 0.05 | 11.15 ± 0.11 | 12.00 ± 0.12 |
| methyl acetate | 0.00± 0.00 | 0.00± 0.00 | 0.00± 0.00 | 0.00 ± 0.00 | 2.56 ± 0.02 | 3.06 ± 0.02 | 3.37 ± 0.02 |
| 2,5-dimethylpyrazine | 0.00± 0.00 | 0.04± 0.00 | 0.02± 0.00 | 0.00 ± 0.00 | 1.75 ± 0.04 | 1.93 ± 0.03 | 2.12 ± 0.07 |
| hexan-1-ol | 0.32± 0.02 | 4.20± 0.04 | 2.64± 0.01 | 0.39 ± 0.02 | 1.24 ± 0.02 | 3.26 ± 0.09 | 3.59 ± 0.03 |
| 2-methylpentan-3-ol | 0.26± 0.02 | 0.81± 0.02 | 0.92± 0.01 | 1.07 ± 0.01 | 1.02 ± 0.02 | 0.85 ± 0.01 | 0.81 ± 0.02 |
| 2-hydroxypentan-3-one | 0.14± 0.01 | 0.46± 0.02 | 0.52± 0.02 | 0.59 ± 0.02 | 0.35 ± 0.01 | 0.31 ± 0.02 | 0.30 ± 0.01 |
| nonanal | 0.21± 0.01 | 0.63± 0.06 | 0.46± 0.01 | 0.21 ± 0.01 | 0.33 ± 0.01 | 0.19 ± 0.01 | 0.21 ± 0.01 |
| 2-Ethyl-6- methylpyrazine | 0.00± 0.00 | 0.02± 0.00 | 0.01± 0.00 | 0.00 ± 0.00 | 0.93 ± 0.01 | 3.27 ± 0.21 | 3.29 ± 0.23 |
| ethyl octanoate | 7.39± 0.42 | 6.77± 0.41 | 6.33± 0.12 | 5.69 ± 0.10 | 4.19 ± 0.04 | 1.03 ± 0.01 | 0.92 ± 0.03 |
| heptan-1-ol | 0.00± 0.00 | 0.71± 0.06 | 0.44± 0.01 | 0.05 ± 0.00 | 0.47 ± 0.00 | 0.85 ± 0.01 | 1.05 ± 0.02 |
| 2-Furaldehyde | 0.10± 0.01 | 0.01± 0.00 | 0.18± 0.01 | 0.45 ± 0.01 | 3.47 ± 0.06 | 4.94 ± 0.06 | 5.53 ± 0.04 |
| 1-(2 methoxypropoxy) propan-2-ol | 1.29± 0.04 | 2.43± 0.11 | 2.98± 0.03 | 3.78 ± 0.03 | 1.46 ± 0.01 | 0.94 ± 0.01 | 0.85 ± 0.01 |
| Benzaldehyde | 1.49± 0.03 | 1.13± 0.06 | 1.13± 0.05 | 1.12 ± 0.01 | 1.40 ± 0.02 | 0.89 ± 0.02 | 0.80 ± 0.01 |
| 2,4-dimethylpentan-3-ol | 0.03± 0.00 | 0.01± 0.00 | 0.03± 0.00 | 0.06 ± 0.00 | 1.02 ± 0.03 | 1.41 ± 0.05 | 1.55 ± 0.06 |
| ethyl decanoate | 6.86± 0.12 | 6.01± 0.21 | 5.66± 0.10 | 5.16 ± 0.08 | 4.16 ± 0.05 | 1.20 ± 0.03 | 1.08 ± 0.02 |
| furan-2-ylmethanol | 0.00± 0.00 | 0.02± 0.00 | 0.03± 0.00 | 0.03 ± 0.00 | 0.71 ± 0.01 | 1.95 ± 0.03 | 2.15 ± 0.03 |
| diethyl  butanedioate | 10.59± 0.60 | 7.63± 0.23 | 6.98± 0.07 | 6.06 ± 0.05 | 5.21 ± 0.03 | 1.24 ± 0.02 | 1.24 ± 0.01 |
| 1-acetyloxydodecyl  acetate | 0.65± 0.03 | 0.21± 0.01 | 0.18± 0.01 | 0.13 ± 0.00 | 0.19 ± 0.01 | 0.00 ± 0.00 | 0.00 ± 0.00 |
| 2-Phenylethanol | 32.17± 1.49 | 18.30± 0.67 | 17.33± 0.22 | 15.94 ± 0.14 | 15.01 ± 0.09 | 4.32 ± 0.06 | 4.75 ± 0.06 |
| 1-methyl-2-propan-2-ylbenzene | 7.94± 0.32 | 2.45± 0.12 | 2.00± 0.08 | 1.36 ± 0.01 | 2.11 ± 0.04 | 0.47 ± 0.03 | 0.47 ± 0.03 |
| 2,5-dimethylfuran | 0.00± 0.00 | 0.00± 0.00 | 0.00± 0.00 | 0.00 ± 0.00 | 0.00 ± 0.00 | 0.87 ± 0.17 | 1.22 ± 0.26 |
| 2,6,6 trimethylbicyclo[3.1.1] hept-2-ene | 0.00± 0.00 | 0.00± 0.00 | 0.00± 0.00 | 0.00 ± 0.00 | 0.00 ± 0.00 | 1.06 ± 0.02 | 1.38 ± 0.03 |
| heptanal | 0.00± 0.00 | 0.00± 0.00 | 0.00± 0.00 | 0.00 ± 0.00 | 0.00 ± 0.00 | 0.55 ± 0.01 | 0.74 ± 0.03 |
| (E)-hept-3-en-2-one | 0.00± 0.00 | 0.00± 0.00 | 0.00± 0.00 | 0.00 ± 0.00 | 0.00 ± 0.00 | 0.42 ± 0.02 | 0.46 ± 0.01 |
| 5-methylfuran-2-carbaldehyde | 0.00± 0.00 | 0.00± 0.00 | 0.00± 0.010 | 0.00 ± 0.00 | 0.00 ± 0.00 | 4.66 ± 0.15 | 5.12 ± 0.14 |

Figure S1 Scoreplot of principal component analysis (PCA), computed on destructive data.


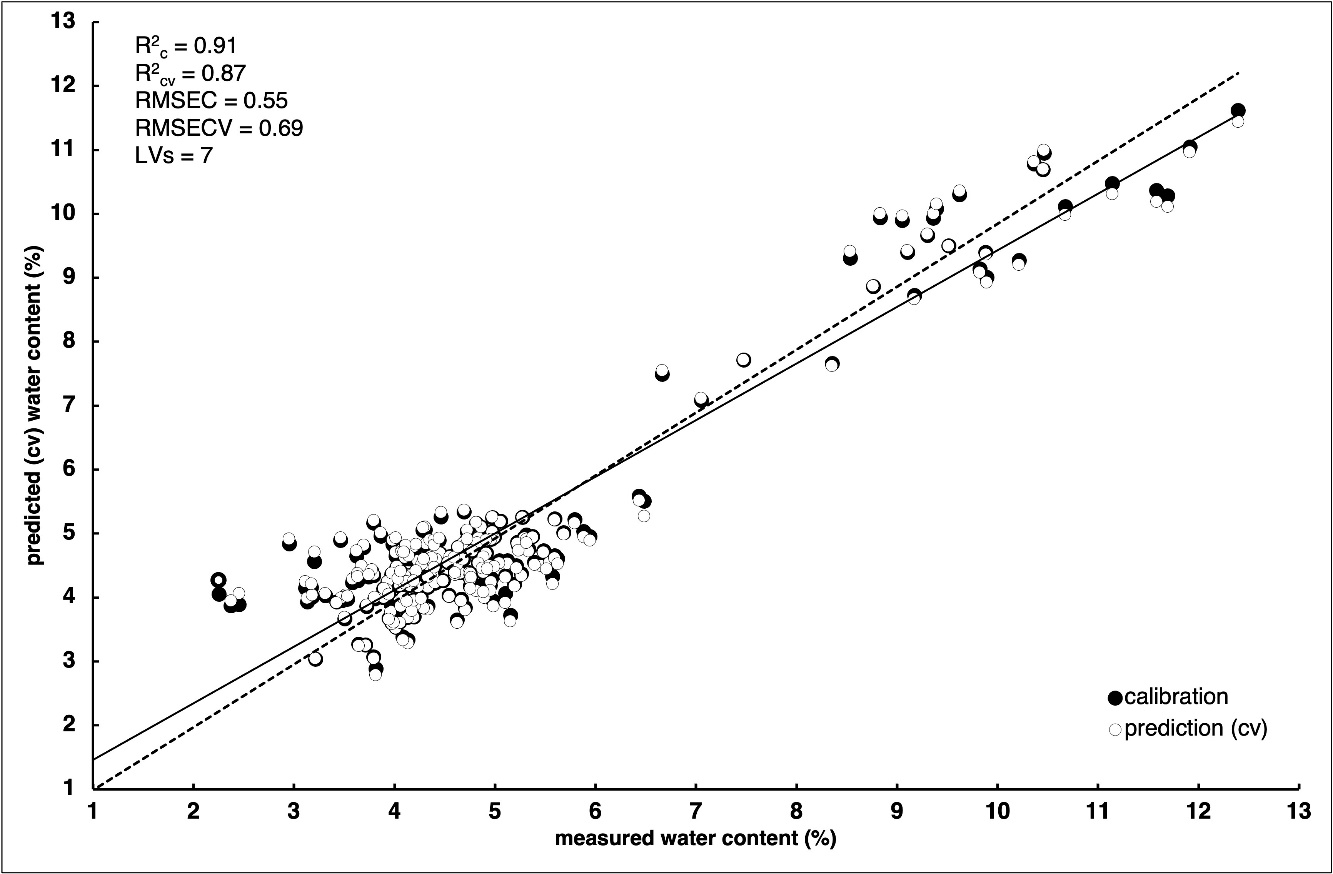


Figure S2 Scatterplot relative to partial least squares (PLS) regressive model for moisture content computed on detected FT-NIR spectra.
